# Interspecies Translation of Disease Networks Increases Robustness and Predictive Accuracy

**DOI:** 10.1371/journal.pcbi.1002258

**Published:** 2011-11-03

**Authors:** Seyed Yahya Anvar, Allan Tucker, Veronica Vinciotti, Andrea Venema, Gert-Jan B. van Ommen, Silvere M. van der Maarel, Vered Raz, Peter A. C. ‘t Hoen

**Affiliations:** 1 Center for Human and Clinical Genetics, Leiden University Medical Center, Leiden, The Netherlands; 2 Center for Intelligent Data Analysis, School of Information Systems, Computing and Mathematics, Brunel University, Uxbridge, Middlesex, United Kingdom; New York University, United States of America

## Abstract

Gene regulatory networks give important insights into the mechanisms underlying physiology and pathophysiology. The derivation of gene regulatory networks from high-throughput expression data via machine learning strategies is problematic as the reliability of these models is often compromised by limited and highly variable samples, heterogeneity in transcript isoforms, noise, and other artifacts. Here, we develop a novel algorithm, dubbed Dandelion, in which we construct and train intraspecies Bayesian networks that are translated and assessed on independent test sets from other species in a reiterative procedure. The interspecies disease networks are subjected to multi-layers of analysis and evaluation, leading to the identification of the most consistent relationships within the network structure. In this study, we demonstrate the performance of our algorithms on datasets from animal models of oculopharyngeal muscular dystrophy (OPMD) and patient materials. We show that the interspecies network of genes coding for the proteasome provide highly accurate predictions on gene expression levels and disease phenotype. Moreover, the cross-species translation increases the stability and robustness of these networks. Unlike existing modeling approaches, our algorithms do not require assumptions on notoriously difficult one-to-one mapping of protein orthologues or alternative transcripts and can deal with missing data. We show that the identified key components of the OPMD disease network can be confirmed in an unseen and independent disease model. This study presents a state-of-the-art strategy in constructing interspecies disease networks that provide crucial information on regulatory relationships among genes, leading to better understanding of the disease molecular mechanisms.

## Author Summary

The identification of gene regulatory networks can provide vital information on biological processes. Despite numerous advancements in developing machine learning strategies, the stochastic nature of such biological systems complicates the construction of robust and reliable network structures. In recent years, the use of cross-species datasets enabled scientists to better understand the molecular mechanisms that are associated with human disorders. However, it also presents a challenge in dealing with especially difficult mapping of protein orthologues, alternative transcript splicing, noise, or other artifacts. Here, we developed a novel algorithm for constructing interspecies disease networks that provide accurate predictive value over the disease phenotype and gene expression. We show that the disease-association of potential key regulators that play a role in interspecies disease networks can be reproduced and validated in an unseen and independent model system. This study presents a novel strategy for constructing networks that can be translated across species whilst providing a comprehensive view of regulatory relationships associated with the disease.

## Introduction

The degree to which gene products appear in the cell and exert their function is regulated through interactions with other genes. This interconnectivity implies that the identification of gene regulatory networks is vital for understanding the phenotypic impacts of gene defects and the associated complications [1]–[4]. The dawn of high-throughput technologies such as genome-wide sequencing and microarray experiments has increased our understanding of molecular behavior at the transcriptional level. Although these large-scale datasets provide crucial information about both the presence and relative abundance of RNA transcripts, they also introduce an important challenge in providing a comprehensive view of molecular mechanisms and regulatory relationships among genes with different underlying phenotypic conditions.

The presence of this obstacle calls for developing robust machine learning models that can be used for generating gene networks in which their transcriptional changes can affect phenotypic outcome. However, building a network that involves thousands of genes and millions of interactions is extremely problematic and requires a great quantity of experimental data for the valid interpretation of biological causes for a given phenotype. Furthermore, the validity of gene regulatory networks is often affected by limited and highly variable samples, heterogeneity in transcript isoforms, noise and other artifacts [5]–[8]. Therefore, a probabilistic approach is needed to identify and predict interconnected transcriptional behaviors that give rise to disease outcome [9] and to, ultimately, offer potential targets for therapeutic intervention and drug development. Among the possible statistical models, Bayesian networks have been an important concept for modeling uncertain systems [10]–[13]. Bayesian networks can represent complex stochastic relationships between genes and are capable of integrating different types of data (i.e. phenotype and genotype categorical information as well as gene expression data). In addition, the probabilistic nature of such networks can accommodate noise and missing data by weighting each information source according to its reliability. In contrast to many statistical models, the transparent nature of Bayesian networks (in terms of the graphical structure and local probability distributions) leads to better interpretation and understanding of the underlying biological regulation of the disease.

The high dimensionality of the genome wide expression profiling datasets and the limited number of available samples complicates the derivation of robust network structures. Methods such as the use of prior knowledge about biological interactions [13]–[15] have been shown to successfully reduce the search space and to make networks more robust. This method works for well-studied diseases or biological systems, but is not likely to identify novel regulatory interactions underlying the molecular mechanisms of rare or complex disorders. In addition, this bias can falsely expose the network to sample differences in the absence of a disease-related biological cause. In this study, we hypothesize that biologically relevant relationships between genes are often conserved across species. Thus, the robustness and stability of a gene network should increase when modeling regulatory networks using related datasets from different species. Moreover, we hypothesize that the relationships identified in an interspecies gene network should be biologically more meaningful. On the other hand, cross-species translation of networks is far from trivial given our limited knowledge of true protein orthologues and transcript variants coding for proteins with similar functions in different species. Therefore, we explore the performance of a novel algorithm that combines our previously published model for learning regulatory interactions from multiple datasets of increasing complexity [16] with an interspecies translation and validation regime, named *Dandelion algorithm*. We show that the supplementation of this algorithm with a modeling-driven selection of transcripts coding for orthologous proteins (*exhaustive* Dandelion algorithm) significantly improves the *robustness* and stability of the *interspecies network*, when compared to a standard approach in which expression levels of different transcripts for the same gene are summarized (*naïve* Dandelion algorithm). We also show that the potential regulatory relationships that play a role in *interspecies disease networks* can be reproduced and validated in an unseen and independent model system.

In this study, three publicly available microarray datasets from *Drosophila* [17], mouse [18], and human [19] that are all concerned with oculopharyngeal muscular dystrophy (OPMD) have been chosen to gain insight into the key regulators of the disease. These datasets are described in Table 1. OPMD is a late-onset progressive muscular disorder for which the underlying molecular mechanisms are largely unknown. This autosomal dominant muscular disorder has an estimated prevalence of 1 in 100,000 worldwide [20]. OPMD is caused by the expansion mutation of a homopolymeric alanine stretch at the N-terminus of the Poly(A) Binding Protein Nuclear 1 (PABPN1) by 2–7 additional Ala residues [21]. Although PABPN1 is ubiquitously expressed, the clinical and pathological features of OPMD are restricted to a subset of skeletal muscles, causing progressive *ptosis*, *dysphagia*, and limb muscle weakness. *Drosophila* and mouse models with muscle-specific overexpression of expanded PABPN1 recapitulate progressive muscle weakness in OPMD [22], [23]. However, the potential artifact, heterogeneity in transcript isoforms, and the presence of overexpression side-effects in OPMD animal models and limited patient materials complicate the identification of key regulators of OPMD. With the analysis of these datasets, we demonstrate that modeling of *interspecies disease networks* increases the *robustness* of the networks and aids in the identification of key regulators of the disease.

**Table 1:** Overview of microarray datasets and networks constructed by Dandelion algorithm.

						Cross-Validation	Number of Networks		
Species	Tissue	Samples	Age/Time-Point	Platform	GEO Accession	Number of folds	Human	Mouse	Drosophila
**Human**	Quadriceps	4 Symptomatic 18 Controls	49–60 Year-old 17–89 Year-old	Illumina 48K Human v.3 Bead Array	GSE26605	4	4	24	24
**Mouse**	Quadriceps	17 OPMD 16 Wild-type	3 Time-Points 6, 18, 26 week-old	Illumina 48K Mouse v.1 Bead Array	GSE26604	6	24	6	36
**Drosophila**	Adult thoracic muscles	18 OPMD 18 Wild-type	3 Time-Points 1, 6, 11 day-old	15K INDAC Spotted Oligonucleotide Array	-	6	24	36	6

## Methods

### Model of Interspecies Networks Using Dandelion Algorithm

**Figure 1:**
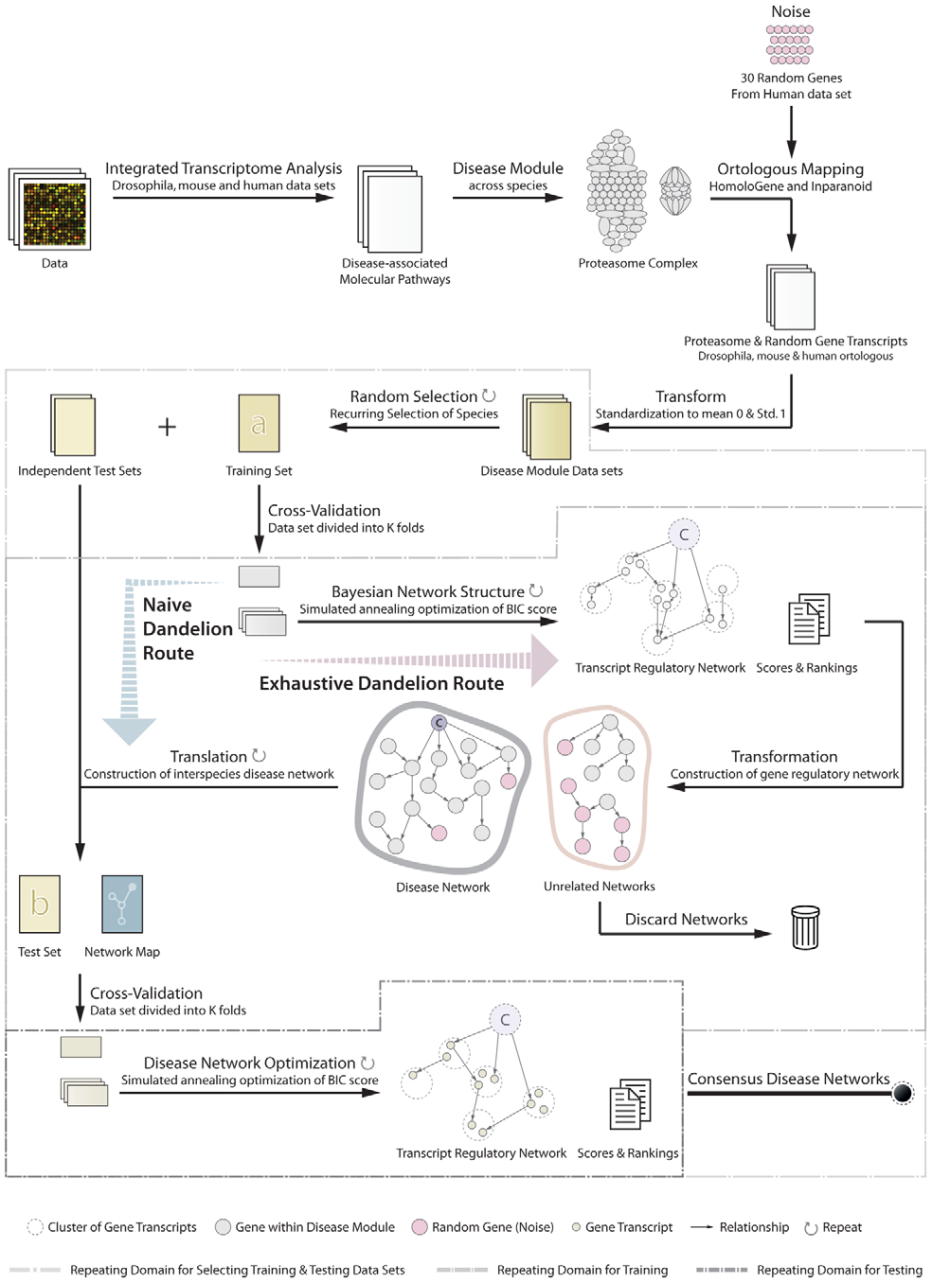
Schematic overview of the Dandelion algorithm for disease network analysis. The Dandelion algorithm involves three recurring stages of training and independent testing regime with the use of multiple datasets derived from different species. In the first step, disease modules are defined as the most consistently disease-associated molecular pathway across species. The disease module is supplemented by a set of randomly selected genes to assess the performance of the algorithm and to check for overfitting. These datasets are standardized to mean 0 and standard deviation of 1 across genes. The next step involves reiterative selection of one species as an organism in which the gene regulatory network is constructed while others are left aside for independent testing and validation of learnt disease networks. For an intraspecies construction of disease network, dataset is divided into k-folds, using cross-validation, and regulatory relationships between gene transcripts are learnt using Bayesian network methodology enhanced by simulated annealing optimization of network BIC score. After applying confidence thresholds on relationship between genes, the disease network can then be translated to the expected interspecies disease network which we call a network map. Using the cross-validation and network optimization procedure the algorithm searches through the relationships found in the training dataset to find the best fit for interspecies representation of the disease network. These networks are then integrated by removing all the links with low confidence score across species.

To construct *interspecies networks* that can accurately predict the disease phenotype and provide a comprehensive view of molecular relationships that underlie the disease-associated biological processes, we developed a novel *Dandelion algorithm* with multi-layers of analysis and evaluation criteria. A schematic presentation of this approach can be found in Figure 1. In addition, the definition of nomenclatures (italicized terms) used in this study is provided in the Table S1 in Text S1.

The procedure starts with the identification of the disease-associated modules by assessing the association of transcriptional profiles with the disease state. In this study, gene modules are defined according to current KEGG (Kyoto Encyclopedia of Genes and Genomes) annotation of molecular pathways to ensure functional relationships among genes within the same cluster. After identification of the *disease module*, the set of genes in the *disease module* is supplemented with a set of randomly selected genes for the purpose of network performance estimation and evaluation. The Dandelion algorithm integrates three recurring phases of training and independent testing with the use of multiple datasets derived from the different biological systems. This involves a reiterative selection of one species as an organism in which *intraspecies* gene regulatory networks are constructed. Cross-validation is used for learning and optimization of the intraspecies network structure. Some partitions were purely used for testing the intraspecies network to ensure, in all experiments, that the test data is previously unseen. Datasets from the other species are used for *interspecies* translation, independent testing and validation of the constructed disease networks. The construction of intraspecies Bayesian networks is governed by our previously published optimization procedure [16]. To ensure that these *interspecies networks* are derived from a disease-related biological cause, the *specificity* and *sensitivity* of the networks for prediction of the disease phenotype are assessed. Moreover, the *robustness* and *translatability* at different *confidence* thresholds are evaluated. After defining the *interspecies disease domains*, a subset of genes is selected for unbiased examination of reproducibility and validity of disease-related transcriptional changes in an unseen and independent model system. The detailed outline of the procedure, depicted in Figure 1, is provided in the following subsections.

#### Disease modules

Disease modules have been identified according to our previously published study [19] in which we performed an integrated transcriptome analysis to identify the most significant molecular pathways that are associated with the OPMD across species.

#### Bayesian network structure learning

A Bayesian network encodes the joint probability distribution of a set of random variables. It consists of a directed acyclic graph (DAG) that represents conditional independencies between variables, and conditional distributions at each node in the graph. Bayesian network classifiers are a special case of Bayesian networks where one node represents some discrete class to be predicted. Here, each node in the graph represents a gene transcript (or gene) and the class node represents the disease states. In order to learn the Bayesian network structure of a gene network, the algorithm approximates the likely graphical model by searching the space of possible networks via single-arc changes that improves some score. We use a simulated annealing search in conjunction with the Bayes Information Criterion (BIC) as a scoring metric [24]. Simulated annealing performs competitively with other optimization methods as it aims to avoid local maxima [25]. There is a trade-off between simplicity of model with one that can accurately identify the empirical distribution of gene expression profiles and predict the disease phenotypic outcome. For this reason the BIC is used as it is less prone to overfitting through the use of a penalizing term for overly complex models.

The initial state of the structure is an empty DAG with no links. In order to alter the network structures, three operations have been used within the simulated annealing procedure. These operators are *adding*, *removing*, or *swapping* links to generate a new network which can be either accepted or rejected based on its overall score and the current temperature. The outline of this algorithm can be found in the Protocol S1 in Text S1.

In this study, the initial temperature (*t_0_*) has been set to 10 and it terminates at 0.001 (*t_n_*), according to our previously published optimization procedure [16]. The number of iterations (*maxfc*) has been set to 1000 in respect to the number of nodes available in the network. The training dataset is described as *D*. For the training phase, the *mode* variable is set to “train” and the variable *networkMap* is set to empty. During the interspecies translation and testing, the variable *mode* is set to “test” and the variable *networkMap* holds information on the regulatory relationships that are present in the network map constructed on training organism.

#### Construction of interspecies networks

The Dandelion algorithm takes multiple datasets from different species as input. In this study, we launch two classes of Dandelion algorithm. Firstly, the naïve Dandelion algorithm, where the expression patterns of gene transcripts are summarized by averaging the expression profiles of gene probes, to provide one expression profile per gene. This enables direct mapping of expression profiles of orthologous genes when translating networks across species. This approach significantly simplifies the process of constructing network structures. Secondly, we developed the exhaustive Dandelion algorithm to overcome the limitations caused by heterogeneity in transcript isoforms, differences in annotation between organisms and technical factors (i.e. different microarray platforms). In the exhaustive algorithm, transcripts that are most likely to be coding for orthologous proteins are selected automatically in the modeling phase.

The procedure involves reiterative selection of one species for construction of the Bayesian network while other species are left aside for independent testing and validation of learnt disease networks. The highest-scoring intraspecies network structure is learnt according to the algorithm described in the Protocol S1 in Text S1. Before interspecies translation, in the exhaustive Dandelion algorithm, a detailed interaction map of a candidate intraspecies disease network of gene transcripts needs to be transformed to a network map of gene-gene relationships. This step can be omitted in the naïve Dandelion algorithm as the constructed intraspecies networks are already at the gene level. Using the cross-validation and network optimization procedure, the algorithm searches through the relationships present in the network map (constructed on the training set) to find the best fit for the interspecies representation of the disease network. These networks are then integrated by removing all the links with a low confidence score to construct the consensus interspecies disease networks. The full algorithm details are outlined in the Protocol S2 in Text S1 where *Species_train_* and *train_folds_* represent the training dataset and the folding arrangements for the selected organism. Furthermore, the series of *Species_test 1_* … *Species_test M_* and *test_folds 1_* … *test_folds M_* represent the datasets and folding arrangements of organisms that are selected for independent test and validation. The logical variable exhaustive indicates the class of Dandelion algorithm (naïve in case of *false* and exhaustive in case of *true*) that needs to be performed.

In this study, the human dataset is divided into 4 folds due to the limited number of patient samples. Mouse and *Drosophila* datasets are divided into 6 folds. The average *Sum of Squared Error* (SSE) and standard deviation (STD) are calculated for all nodes over these folds by predicting the measured expression values of genes (or gene transcripts) given the measurements taken from others. For the class node, the state of the disease is predicted given the expression profiles for genes (or gene transcripts) within the network structure. The number of iterations was set to 1000 for the training phase and was reduced to 500 during the interspecies translation of disease networks. The code is implemented in Matlab 2008b using the Bayes Net toolbox [26].

#### Network analysis and evaluation

The proposed approach consists of three layers of analysis and evaluation. The constructed interspecies disease networks are assessed for their predictive accuracy towards the disease phenotype (class node) by calculation of the level of sensitivity and specificity. Furthermore, the Bayesian networks Sum of Squared Error (SSE) is calculated for prediction of the expression of all genes (or gene transcripts). Moreover, the level of robustness and translatability of the generated networks are evaluated. The stability and robustness of relationships between genes within the disease module are compared to those of the random genes at different confidence score thresholds. Confidence scores are the ratio of the number of times a link is found in the interspecies disease networks to the maximum number of times the link can possibly be found (based upon the number of folds). For approximating the level of translatability, the total number of links found during the training phase is compared to the number of links that were successfully translated to other species. Finally, the interspecies disease domains are defined based on the Markov blanket principle for the extension of the class node connectivity. In addition, unstable gene interactions are removed through assessment of the level of confidence in the relationships between genes. The interspecies disease domains are used to select a subset of genes to further study the reproducibility and validity of the observed relationships towards their association with the disease phenotype in an unseen and independent OPMD model system.

To assess the specificity of genes encoding for the proteasomal proteins in accurately predicting the disease states, we generated three additional gene sets. A set of 100 randomly selected genes, 87 genes within the ribosome pathway, and 70 randomly selected genes with the constraint of none being deregulated (ND) constitute the three genes sets that are used in a comparative analysis. The human dataset is used for cross-validation whilst mouse and *Drosophila* datasets were used for independent assessment of the constructed networks. Networks are evaluated on their sensitivity, specificity, and predictive accuracy towards the disease state (OPMD or control).

### Microarray Datasets

The human, mouse, and *Drosophila* microarray datasets have been previously published [17]–[19]. The human and mouse datasets are publicly available at GEO repository under the accession numbers GSE26605 and GSE26604, respectively. In all datasets genome-wide expression profiles of skeletal muscles from OPMD are compared to controls. In case there are multiple probes for the same gene on the microarray platforms, these probes usually measure the expression levels of different transcripts from the same gene. The class node reflects the disease phenotype (control or OPMD) of each sample. A detailed description of these datasets can be found in Table 1.

### Data Processing and Statistical Analysis

Microarray measurements were normalized using the quantile method. In addition, these datasets were standardized to mean 0 and standard deviation 1 across the genes. For the scope of this paper, the human proteasome-encoding genes were annotated using illuminaHumanv3BeadID package in R and the mouse and *Drosophila* homologous were annotated using HomoloGene and Inparanoid (http://ncbi.nlm.nih.gov/homologene and http://inparanoid.sbc.su.se, respectively) online databases. Previously published data were used to identify deregulated genes per species [19]. For cross-validation [27], [28] human data were divided into 4 folds (given the limited number of OPMD samples), while the other datasets were divided into 6 folds (**Table 1**). Human, mouse, and *Drosophila* datasets hold 108, 96, and 78 transcripts, respectively, which encode for 74, 56, and 53 genes (including genes encoding for the proteasome and a set of 30 randomly selected genes). The differences are due to limitations of mapping homologous genes or unavailability of expression data for certain genes in a particular species. The gene lists are provided in the Table S2 in Text S1.

### Cell Model

IM2 cells stably transfected with normal (WTA) or expanded PABPN1 (D7E) and were compared to assess the predictive value of the interspecies modeling approach on an unseen OPMD disease model [29]. Exogenous PABPN1 expression is under control of the desmin promoter. IM2 cells were proliferated in DMEM supplemented with 20% fetal calf serum, 0.5% chicken embryo extract, 5 U/ml interferon gamma, at 33C and 10% CO2. Myotube fusion was induced by culturing in DMEM supplemented with 5% horse serum at 37C and 5% CO2 for four days, after which RNA was extracted from three independent cultures.

### Quantitative RT-PCR Analysis

Total RNA was extracted using the TRIZOL reagent (Invitrogen) according to manufacturer's instruction. First strand cDNA was synthesized with random hexamer oligonucleotides and MMLV reverse transcriptase (First Strand Kit; Fermentas, according to manufacturer's instruction). 3.6 ng cDNA was used per quantitative PCR reaction. qPCR was performed with SYBR green mix buffer (BioRad) and 7.5 pmole (per reaction) of forward and reverse primers in a 15 µL reaction volume. PCR conditions were as follows: 4 min at 95°C followed by 40 cycles of 10 sec at 95°C and 60 sec at 60°C. The program was ended with 1 min at 60°C. For each primer set, the specificity of the PCR products was determined by melting curve analysis. Expression levels were calculated according to the ΔΔCT method normalized to mHrpt, Desmin, and IM2 parental cells. The statistical significance was determined with the student's t-test. The list of primers used in this study is provided in the Table S3 in Text S1.

## Results

### Identification of Disease Module

**Figure 2:**
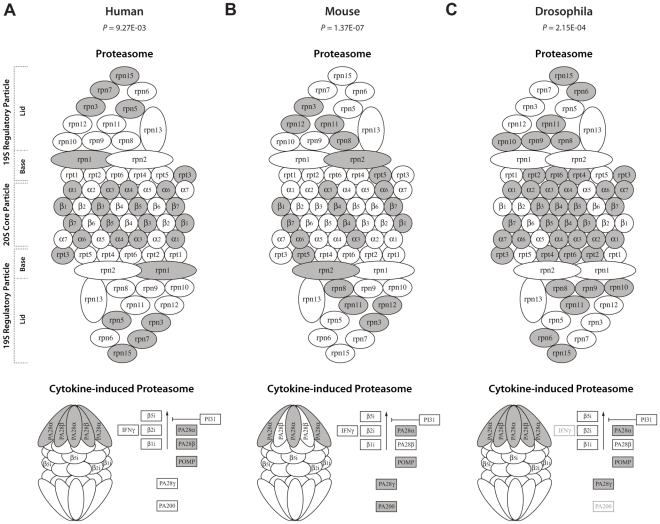
OPMD-deregulation across different subunits of the proteasome in different species. There are widespread differences in gene expression (depicted in dark colors) between OPMD and control in the different functional subunits of proteasome and immunoproteasome in human (**A**), mouse (**B**) and *Drosophila* (**C**). The Significance of the association between the disease outcome and expression profiles of genes encoding for proteasome and immunoproteasome were previously calculated [19] using the global test [46].

Previously we identified that the deregulation of the ubiquitin-proteasome system (UPS) is the predominant molecular pathway affected in OPMD animal models and patients [19]. The UPS, a cellular regulator of homeostasis, is highly dynamic machinery that involves protein ubiquitination and degradation steps. From the six UPS components, we found that only E3-ligases, deubiquitinating enzymes, and proteasome components are consistently and prominently deregulated in OPMD across species [19]. The proteasome is composed of core and regulatory subunits. We observed a substantial deregulation of proteasome and cytokine-induced proteasome (also known as immunoproteasome) encoding genes across species (**Figure 2**). To obtain more insight in the key components in the proteasome machinery that are aberrantly expressed in OPMD across species, we generated gene regulatory networks. Unique to the current approach, the networks were learnt on one species and evaluated on datasets from other species. This was done to only retain those links between genes that can be found across multiple species and that are more likely to be directly connected to the disease phenotype than links that are only found in a single species. For the interspecies translation we used two version of our newly developed Dandelion algorithm. The naïve variant is a straw man approach, where expression values for different transcripts of the same gene are first summarized. This approach was then further refined in the exhaustive Dandelion algorithm, where the model chooses the transcript that is most predictive for the expression value of a transcript in another species.

### Naïve Construction of Disease Network

**Figure 3:**
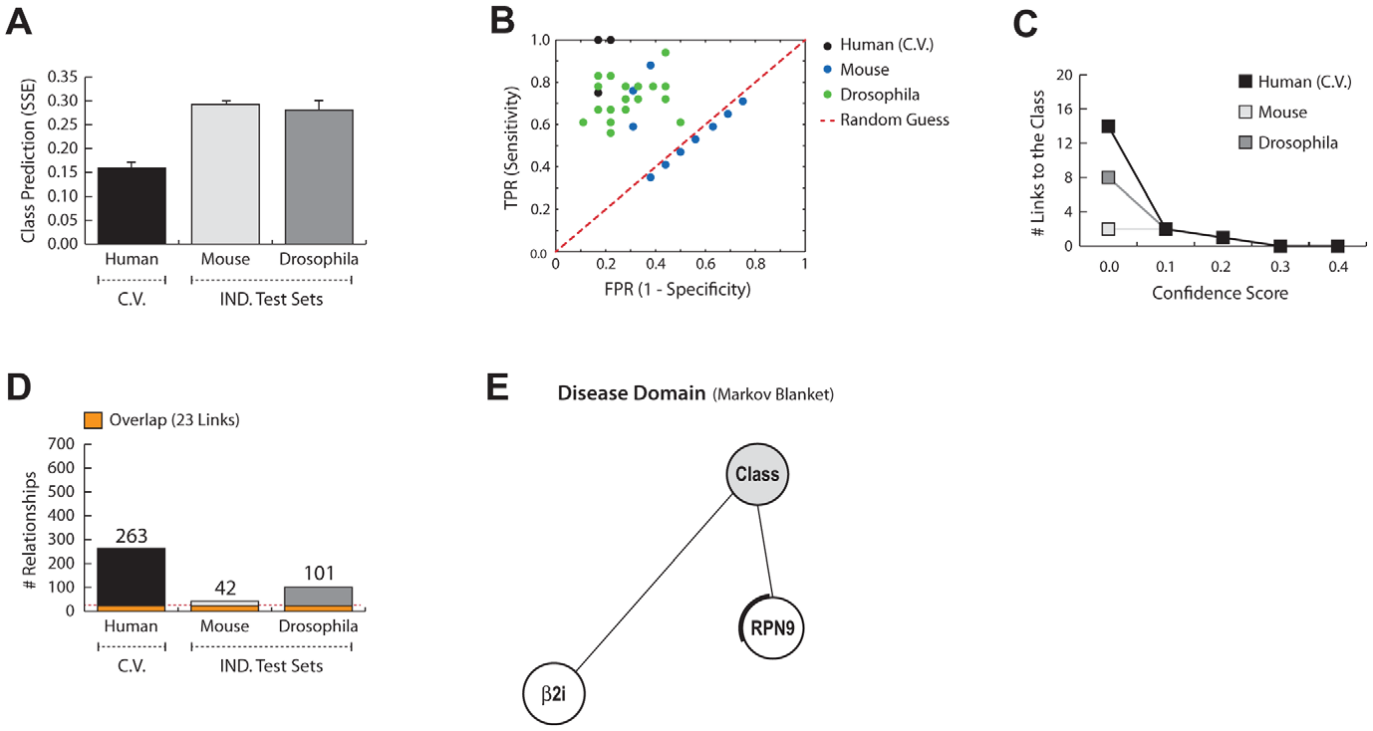
Performance of the naïve Dandelion algorithm on constructing disease networks that are learnt on human and evaluated on human, mouse and *Drosophila* datasets. **A**) The average Sum of Squared Error (SSE) for prediction of the disease phenotype (OPMD vs. control) given the gene expression profiles within the disease networks learnt on human. The cross-validation set which is used during the training phase is depicted by *C.V.* and the independent test sets are grouped as *IND. Test Sets*. **B**) ROC space demonstrates the relative sensitivity and specificity of the generated networks in predicting the disease phenotype. The results from random expectations are illustrated by the red dash-line. **C**) Number of relationships between genes and the class node, after applying confidence thresholds, are depicted in line per species. **D**) The number of links found after interspecies translation and optimization of the disease networks within each species. The orange section, separated by red dash-line, represents the number of links that can be found in all species with the confidence threshold of 0.1. **E**) The interspecies disease domain is generated according to the Markov blanket criteria, after applying the confidence threshold of 0.1.

The process of constructing disease networks using naïve Dandelion algorithm initially starts by averaging the expression profiles of different gene transcripts in the human datasets. The summarized gene expression values were then used for the learning of intraspecies gene networks which consequently were translated to the other species. The interspecies networks were assessed for their predictive accuracy, sensitivity and specificity (**Figure 3**). The constructed interspecies networks predict the disease status (control vs. OPMD) of the unseen *Drosophila* and mouse samples with a moderate accuracy of 71% and 72%, respectively (**Figure 3A**). However, a large number of networks perform worse than random expectations, as evident from the ROC space (**Figure 3B**). This result indicates an overall low level of sensitivity and specificity in predicting the disease phenotype. Moreover, the networks are weak and unstable as they exhibit a very low level of translatability (**Figure 3C**). The low level of robustness, stability and translatability is also evident from the low percentage (8.7%) of relationships with the confidence score of ≥0.1 in the intraspecies networks (**Figure 3D**). Similarly, after applying the confidence threshold of 0.1, the interspecies disease domain structure collapses as only two links survive this constraint (**Figure 3E**). The level of confidence in relationships within the interspecies disease domain is estimated to be between 0.25 and 0.75 for both links and *RPN9* is the only gene found differentially expressed in the *Drosophila* dataset. This indicates that averaging the expression patterns for different gene transcripts reduces the information content of the network considerably and should be avoided for accurate prediction of the disease phenotype and generating biologically relevant regulatory networks.

### Exhaustive Construction of Disease Network

We used the exhaustive Dandelion algorithm to overcome these limitations and provide a detailed interaction map of molecular pathology that extends our knowledge of disease mechanism across species. In contrast to the naïve variant, the exhaustive Dandelion algorithm searches the space of possible relationships at the level of gene transcripts to find the best scoring interspecies regulatory network. It can accommodate missing data and possible dissimilarities by identifying the best fit for a given relationship across species.

**Figure 4:**
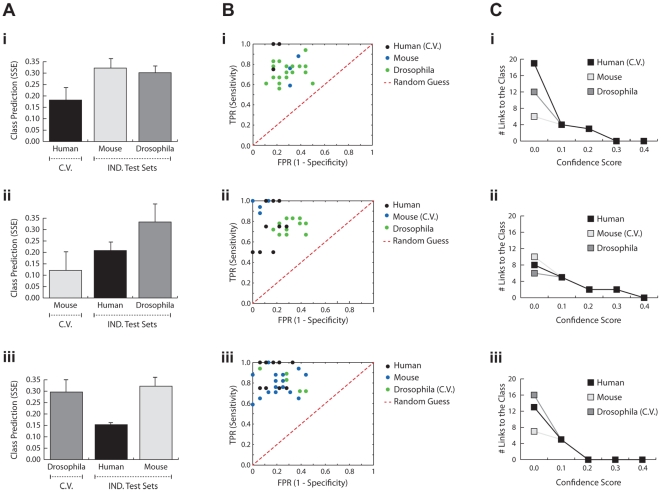
Performance of the exhaustive Dandelion algorithm. **A**) The average Sum of Squared Error (SSE) for prediction of the disease phenotype (OPMD vs. control) given the gene expression profiles within the disease networks learnt on human (**i**), mouse (**ii**), or *Drosophila* (**iii**). The cross-validation set which is used during the training phase is depicted by *C.V.* and the independent test sets are grouped as *IND. Test Sets*. **B**) ROC space demonstrates the relative sensitivity and specificity of the generated networks in predicting the disease phenotype. The results from random expectations are illustrated by the red dash-line. **C**) Number of relationships between genes and the class node, after applying confidence thresholds, are depicted in line per species.

Bayesian networks which are generated using the exhaustive Dandelion algorithm can accurately predict the disease status from the expression levels of genes coding for proteasomal components (**Figure 4A**). We observe over 91% sensitivity and 80% specificity in the prediction of the disease phenotype in the human dataset (with an average SSE under 0.18), and similar values were obtained for the *Drosophila* and mouse datasets. The interspecies disease networks have very high predictive value for other species while they tend to avoid overfitting to a given dataset. This is evident from the low level of variation in SSE between constructed interspecies networks (0.06 in human, 0.11 in mouse, and 0.08 in *Drosophila*). The predictive ability of the interspecies models is highly robust towards the use of different organisms for training and testing, as the average SSE for a given species only slightly varies between different networks. Furthermore, the generated interspecies disease networks exhibit high sensitivity and specificity scores towards their informativeness to the prediction of the disease status. The majority of these networks provide sensitivity and specificity scores higher than 70% (**Figure 4B**). All constructed networks perform significantly better than random expectations, as presented in the ROC spaces (**Figure 4B**). In addition, the gene networks are strongly connected to the class node (representing information on the control and disease states of the samples) since the number of genes connected to the class node only drops to 0 when the confidence threshold was raised to 0.3, 0.4, or 0.2 for networks learnt on human, mouse, or *Drosophila*, respectively (**Figure 4C**). These are very restrained confidence thresholds as they require networks to share the same level of confidence for interactions across all species, and compare favorably to the low number of links remaining at the lower threshold of 0.1 with the naïve Dandelion algorithm.

**Figure 5:**
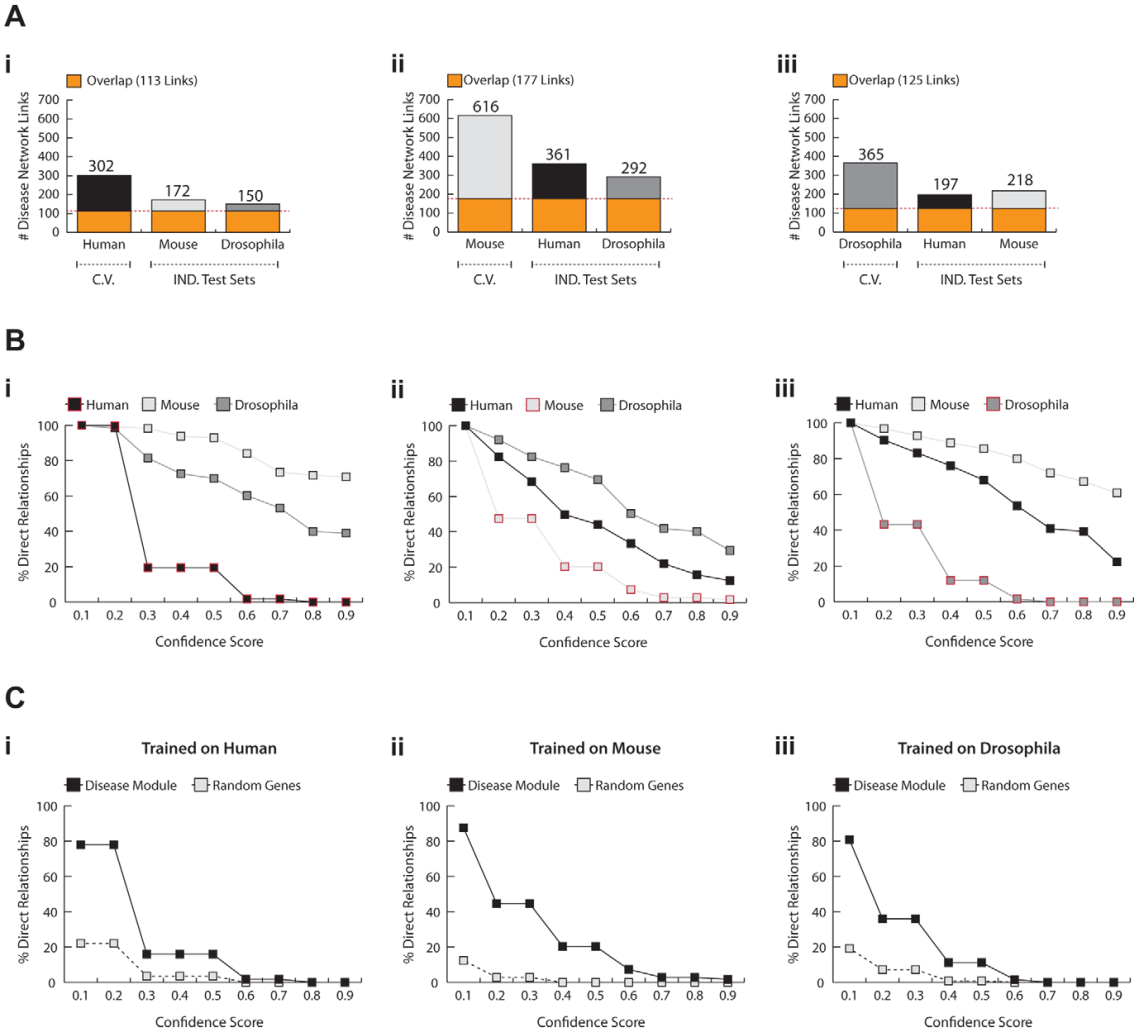
Translatability and robustness of interspecies disease networks. **A**) The number of links that were found during interspecies translation and optimization of the disease networks per individual datasets. The red dash-line depicts the number and fraction of links that can be found in all species with the confidence threshold of 0.1. The translatability of disease networks learnt and trained on human (**i**), mouse (**ii**), and *Drosophila* (**iii**) are presented separately. The cross-validation set which is used during the training phase is depicted by *C.V.* and the independent test sets are grouped as *IND. Test Sets*. **B**) The translatability of relationships over series of different confidence thresholds. These line plots demonstrate the percentage of relationships with confidence score higher than the threshold. For the independent testing datasets the ratio is towards the number of links that were expected to be found after generation of the network map. **C**) The robustness of disease networks are assessed according to the level of connectivity for genes encoding for the proteasome as compared to the set of randomly selected genes at different confidence thresholds.

Figure 5 demonstrates the level of robustness and translatability of the obtained disease networks. A large fraction of relationships (37.4% in human, 28.7% in mouse, and 34.3% in *Drosophila*) can be translated and found in the interspecies disease network with the confidence threshold of 0.1 (**Figure 5A**). Remarkably, an average of more than 60% of the translated links can be found in all organisms. It is evident that the intraspecies networks are highly resistant towards noise and the range of confidence in which interactions can be found in the training set is at least 0.7 and are as high as 0.9 in *Drosophila* and mouse datasets (**Figure 5B**). This value is even higher for relationships that are successfully translated from the intraspecies network to the other organisms (**Figure 5B**). Noticeably, the interspecies networks can still be obtained when applying a very stringent confidence threshold of 0.9 for all three constructed interspecies disease networks. More than 71% and 39% of translated relationships from human pass the confidence threshold of 0.9 in mouse and *Drosophila* datasets, respectively. However, a slightly more severe drop in translatability rate is observed for networks learnt on the mouse data. This can be expected due to the presence of overexpression and possibly other artifacts in this model system, also reflected by the higher level of interconnectivity of these networks. Despite the presence of noise and other artifacts in these datasets, a large fraction of interactions between genes encoding for the proteasome have high confidence scores in the interspecies networks (**Figure 5B**). This is not true for links associated with the randomly selected genes as the majority of those relationships do not pass the confidence threshold of 0.1 (**Figure 5C**). Overall, these results show model-driven selective and predictive ability of the exhaustive Dandelion algorithm in capturing the disease-related relationships between genes in which exhaustive Dandelion significantly outperforms the naïve Dandelion algorithm.

**Figure 6:**
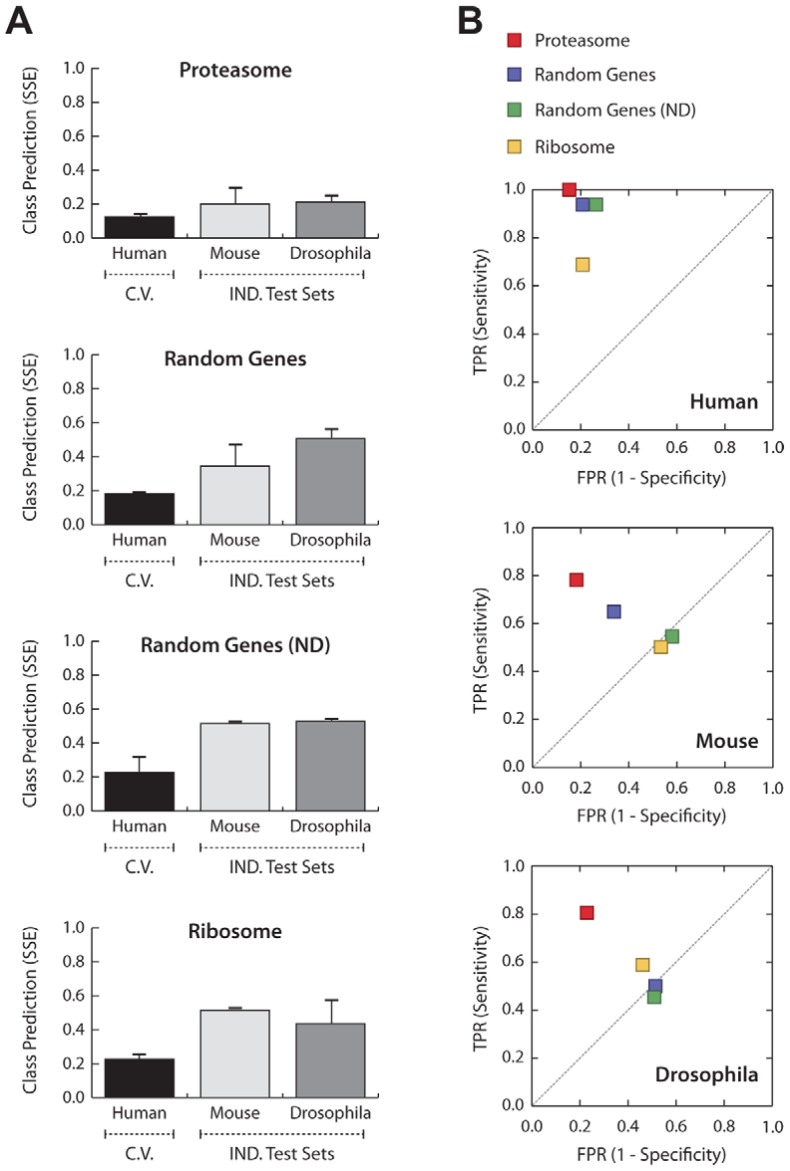
Specificity of the proteasome towards prediction of disease states. **A**) The average Sum of Squared Error (SSE) for prediction of the disease phenotype (OPMD vs. control) given the gene expression profiles within the constructed networks learnt on the proteasome, 100 random genes, 70 not-deregulated random genes (ND), and the ribosome. The cross-validation set which is used during the training phase is depicted by *C.V.* and the independent test sets are grouped as *IND. Test Sets*. **B**) ROC space demonstrates the relative sensitivity and specificity of the generated networks in predicting the disease phenotype. The proteasome, 100 random genes, 70 random genes (ND), and ribosome are illustrated in different colors (red, purple, green, and yellow, respectively). The results from random expectations are illustrated by the gray dash-line.

To assess the specificity of the proteasome in providing accurate prediction of the disease status, we compared the SSE, sensitivity, and specificity of the networks learnt on the proteasome to that of three additional gene sets. The exhaustive Dandelion algorithm was applied to a set of 70 random genes from which none is deregulated (ND) in OPMD, a set of 100 randomly selected genes containing also deregulated genes that are expected to link with the class node in one species but not necessarily across species, and 87 genes coding for the structurally-related ribosomal proteins, which are not known to be consistently differentially expressed in different species [19]. Noticeably, interspecies networks constructed on the proteasome significantly outperformed (86% sensitivity and 81% specificity across species) those constructed on other gene sets (**Figure 6**). Strikingly, the predictive accuracy of networks learnt on the proteasome was slightly improved from the previous experiment (**Figure 4**) in which additional 30 random genes were included. In contrast, the class prediction performance of the other networks was much lower. The class prediction error for networks learnt on the random genes was much higher than that of the proteasomal genes (average SSE of 0.43 and 0.21, respectively) but slightly lower than that of non-deregulated random genes and the ribosome (0.52, and 0.48, respectively) (**Figure 6A**). Although the performance is still acceptable for training and testing on human, the decrease in the level of sensitivity and specificity of non-proteasomal networks is particularly apparent during the translation phase (in this case from human data to mouse and *Drosophila*) (**Figure 6B**), indicating that the links between non-proteasomal genes are not conserved across the different species. Altogether, these results indicate a model-driven selective ability of the algorithm in capturing the most informative and consistent gene relationships which led to the construction of a highly robust interspecies disease network.

### Network Genes and Identification of Key Regulators

**Figure 7:**
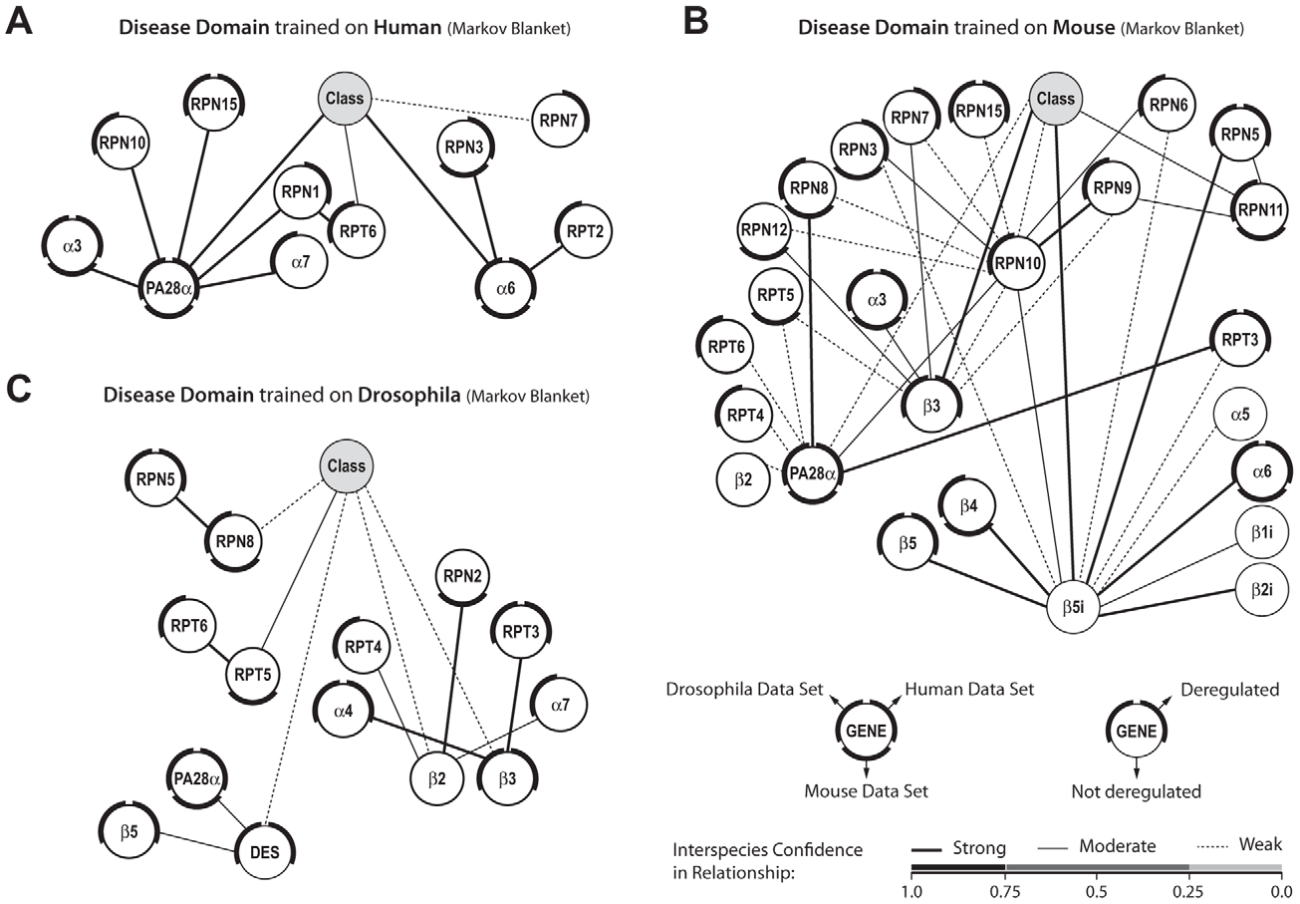
Interspecies disease domains. These interspecies class network structures are learnt on human (**A**), mouse (**B**), or *Drosophila* (**C**) dataset and optimized across species. Class network structures are presented according to Markov blanket criteria. Nodes represent genes. The outer ring reflects deregulation in the expression in the different species (**a**, **b**). Relationships are depicted with lines that represent different degree of confidence in relationships (described in **c**).

Interspecies disease domains represent the most robust, disease-associated gene networks. They are identified by the class node (describing the disease status) and the associated Markov blanket of interactions with the confidence threshold of 0.1 across species (**Figure 7**). In the original experiment, the interspecies disease domain that is trained on human data shows the most robust network as the overall confidence in relationships is very high (**Figure 7A**). The mouse data, however, produced the highest number of relatively weaker relationships among genes (**Figure 7B**). The interspecies disease domain that is trained on the *Drosophila* data shows the same level of robustness as those constructed and trained on human (**Figure 7C**). In *Drosophila*, *Desmin* (*DES*), a randomly selected gene, is connected to the class node as part of the disease domain. Although *DES* (a muscle-specific class III intermediate filament) is a member of the random set, it is significantly deregulated in both human and *Drosophila* datasets. This gene has been clearly linked to muscle differentiation [30] and is likely associated with the OPMD phenotype. No other randomly selected genes appear in the disease network which indicates the reliability and the specificity of the obtained networks. Overall, the interspecies disease domains exhibit a high level of robustness and informativeness towards different states of the disease. This is due to the presence of relationships that can be translated across species with at least a moderate confidence (91.7% in human, 55.3% in mouse, and 71.4% in *Drosophila*). Moreover, the interspecies disease domains contain a large number of nodes that are differentially expressed in at least one species (100% in human, 80% in mouse, and 92.9% in *Drosophila*). Furthermore, the majority of genes are shared between at least two interspecies disease domains (81.8%, 64%, and 78.6%, for disease domains after training on human, mouse and *Drosophila*, respectively). Many of the links between genes present in these network structures demonstrate a strong correlation in expression profiles in the different species (**Table S4** in **Text S1**). Overall, these results indicate that the expression levels of the majority of genes in the constructed interspecies networks are strongly correlated and more likely to be associated with the OPMD phenotype than genes that are differentially expressed in single species.

### Evaluation of Disease Networks on Unseen Disease Model

**Figure 8:**
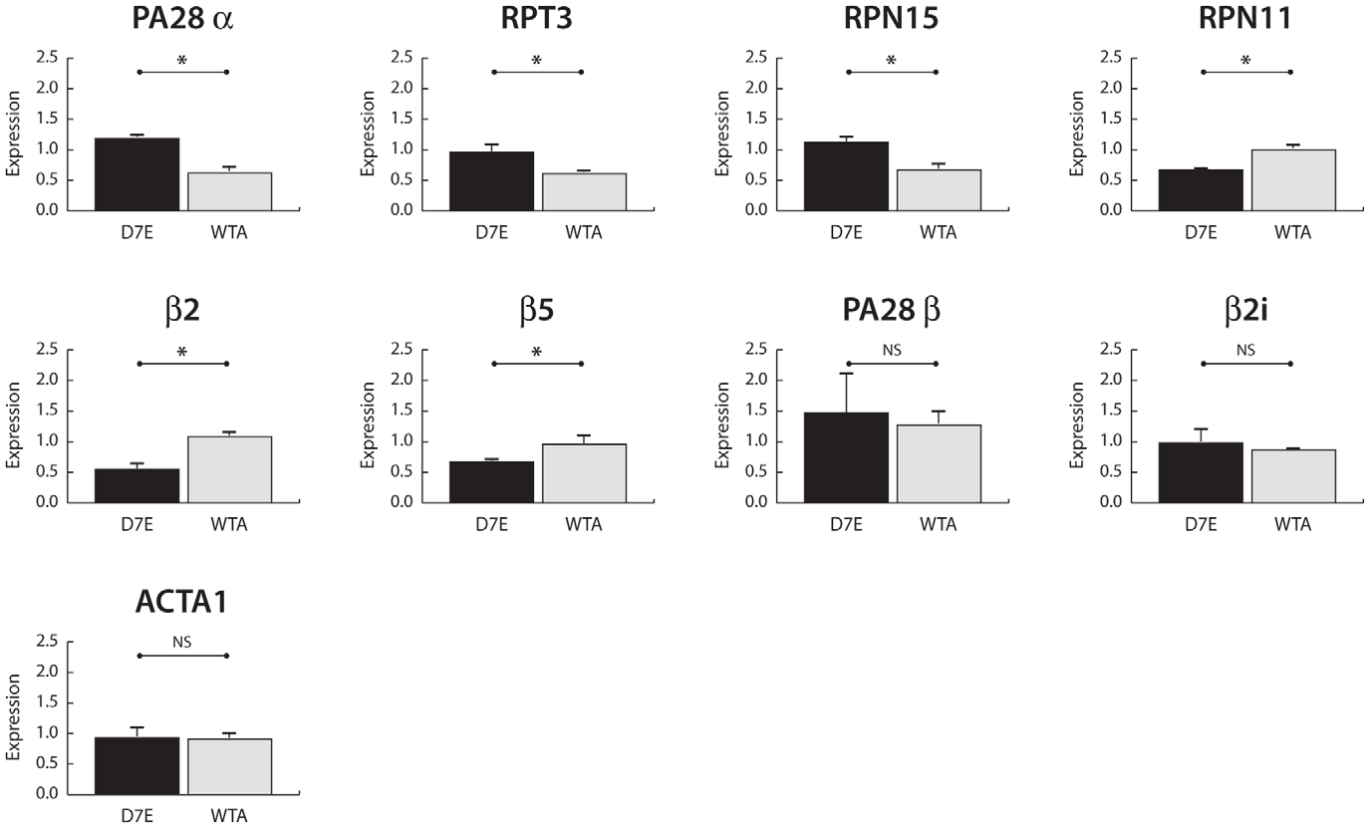
Validation of differential expression of disease associated genes in an unseen disease model. Results from qPCR experiments measuring differences in gene expression between control cells (WTA, N = 3 independent cultures) and cells expressing the OPMD-associated PABPN1 with expanded repeat (D7E, N = 3 independent cultures). Expression levels were normalized to *Desmin* to correct for differences in the myogenicity in the different cell cultures. Significant differences (*P*<0.05, Student's T-test) between measured expression values in D7E and WTA cells are indicated by *, whilst NS stands for no significant difference. *PA28α*, *RPT3*, *RPN15*, *RPN11*, *β2*, and *β5* expression in IM2 cell lines were selected from the group of genes present in the interspecies disease domain. *PA28β* (deregulated in human dataset) was selected as its role in assembling the lid subunit of the immunoproteasome is highly similar to *PA28α* but not part of the interspecies disease domain. *β2i* is one of the two genes that remained connected to the class node in the interspecies disease domain constructed by naïve Dandelion approach. ACTA1 is a control for myotube formation.

The model-driven and interspecies selection of genes that are most likely to be associated with the disease phenotype suggests their association with the disease in an independent and unseen disease model. Therefore, we evaluated the disease-related transcriptional changes for a subset of genes (selected from the interspecies disease domains) in the IM2 cell model [29] with moderate overexpression of the wild-type PABPN1 (WTA) or the mutant PABPN1 protein isoform (D7E). Remarkably, all the selected genes (*PA28α*, *RPT3*, *RPN15*, *RPN11*, *β2*, and *β5*) showed significant differential expression in an unseen IM2 cell model (**Figure 8**). *PA28α* appears to be an essential hub in the interspecies disease domains trained on the human and mouse datasets (**Figure 7**). Noticeably, it is also significantly deregulated between D7E and WTA (**Figure 8**). In contrast, *PA28β*, which is a closely related homolog in the PA28 complex [31] and also significantly deregulated in human dataset, do not play a part in the interspecies disease domains. Interestingly, it is evident that the expression pattern of *PA28β* is not deregulated between the D7E and WTA cells (**Figure 8**). Next, we assessed the expression of the *β2i*, a member of immunoproteasome core subunit, present in the interspecies disease domain constructed with the naïve Dandelion algorithm. This gene is not differentially expressed between D7E and WTA cells (**Figure 8**). Overall, these results highlight the unique ability of the exhaustive Dandelion algorithm to identify disease-related genes that can be found across different OPMD model systems and patients.

## Discussion

Integration of transcriptome data from different species is far from trivial and is complicated by our limited knowledge of true protein orthologues and transcript variants coding for proteins with similar functions. Moreover, the presence of noise and artifacts specific to certain model systems usually leads to limited overlap between results obtained in cross-species comparisons [32]–[35]. In this paper, we developed a Bayesian-based methodology (Dandelion algorithm) to model gene networks associated with the same disease in different species. We showed that the integration and analysis of gene expression datasets from various species increase the robustness of the constructed networks and the predictive accuracy of the disease state. We also demonstrated that the interspecies translation of the networks helps to avoid overfitting. A newly developed model-driven selection of transcripts that are most likely to be coding for orthologous proteins is essential for the generation of robust interspecies disease networks.

Our approach for Bayesian modeling of datasets on a similar phenotype from different model systems and patients is rather unique. Several approaches have been described to avoid overfitting and increase the robustness of Bayesian networks. For example, informative priors derived from protein-protein interaction (PPI) data or from the literature have been used to generate more stable and biologically meaningful networks [13]–[15], [36]. While these methods obviously bias the results towards well-known regulatory interactions [37], [38], these methods may ultimately be combined with our modeling approach to obtain regulatory networks with a more straightforward biological interpretation.

Our method was applied to an *a priori* defined gene module coding for a well-known biological structure, the proteasome. Several studies in *S. cerevisiae* [39]–[42] have demonstrated the value of an integrative modeling approach providing modularized interaction networks without prior assumptions. Zhang et al. [39], for instance, took an approach in which they integrated a number of different available data sources, from PPIs to sequence homology and gene co-expression, while Tanay et al. [40] and others [41], [42] expanded on the statistical analysis of network properties and identifying modules within the network structure. The performance of these models depends on the availability of high quantities of samples and may be prone to overfitting due to the presence of noise and other model-specific artifacts. Therefore, a combination with our interspecies translation approach may enable the allowing of larger gene regulatory networks with multiple gene modules and connections between them.

In this study, three microarray datasets from *Drosophila*, mouse and human, that are all concerned with OPMD, are used to gain insight into key regulatory relationships of interspecies disease networks that are directly and robustly associated with the disease. Previously, we have established the importance of the deregulation of the ubiquitin-proteasome system (UPS) for the disease etiology [19]. From the different components of the UPS, the down-regulation of the proteasome has been associated with the late-onset of the disease [19] as the reduced proteasome activity can lead to futile protein degradation. However, little is known about the key components of the proteasome that are contributing to the OPMD phenotype. Hence, the generation of interspecies disease networks for the proteasome encoding genes now shed some light on the underlying regulatory mechanisms that govern the disease-related transcriptional changes of the proteasome encoding genes.

We identified PA28α, one of the three components of the PA28 subunit, as an important hub gene in the interspecies disease domain and validated its significant differential expression in an unseen disease model. PA28α plays an important role in assembling the lid subunit of the immunoproteasome and stimulating the proteasome core component [31]. Previously we showed that the induction of immunoproteasome activity leads to a significant reduction in the nuclear expPABPN1 accumulation [19]. This observation further signifies the role of PA28 assembly and the immunoproteasome in the disease etiology. In contrast, the other PA28 component PA28β although significantly deregulated in human OPMD patients, appears to play a less crucial role since its association with the disease did not translate to the OPMD animal models and could not be reproduced in the OPMD cell model system. On the other hand, the association of β2 and β5, members of the proteasome core subunit, with the disease was identified by the interspecies disease domains and reproduced in the OPMD cell model. Down-regulation of the proteasome core subunit can lead to futile protein degradation which results in protein accumulation. Our analysis suggests that β2 and β5 are vital regulators of the proteasome activity which are disease associated. It has been shown that the down-regulation of the proteasome core subunit can trigger expPABPN1 accumulation and play a role in the disease late-onset [19]. Relevant to the late-onset of the OPMD, previously it has been shown that the proteasome activity declines during muscle ageing [43]–[45], a phenomena which is highly associated with the transcriptional changes of the proteasomal genes [45]. In follow-up studies, the functional role of proteasomal protein dysregulation in the disease pathology and ageing of muscles needs to be investigated. Furthermore, the functional relevance of gene regulatory relationships should be investigated where changes in protein level mimic the *in vivo* situation and directly affect the protein catabolism. This would ultimately result in better understanding of the mechanism in which the loss of proteostasis leads to degenerative loss of muscle function during ageing and in OPMD.

In conclusion, this study presents a state-of-the-art strategy in constructing interspecies disease networks that provide crucial and comprehensive information on gene regulatory relationships. This leads to better understanding and identification of the molecular mechanisms underlying the disease. The high level of specificity and sensitivity of these models enables the prioritization of candidate regulators of molecular disease mechanisms to be studied in follow-up validation experiments. In particular, it is crucial to carry out additional experiments to investigate the functional relevance of proteasomal proteins dysregulation to the OPMD pathology. We believe that robust and unbiased construction of the interspecies networks for rare or complex human diseases can lead to novel discovery and identification of key regulators which can ultimately offer potential targets for therapeutic interventions and drug developments.

## Supporting Information

Text S1 This supplementary material includes information on terminological definitions, protocols, gene lists, a set of primers used for validation study, and correlation analysis.
(PDF)
