# Correction: Interspecies Translation of Disease Networks Increases Robustness and Predictive Accuracy

**DOI:** 10.1371/annotation/fc0b4192-6427-4fb3-b347-c66651adf855

**Published:** 2011-11-08

**Authors:** Seyed Yahya Anvar, Allan Tucker, Veronica Vinciotti, Andrea Venema, Gert-Jan B. van Ommen, Silvere M. van der Maarel, Vered Raz, Peter A. C. ‘t Hoen

The following information was missing from the Funding section: This study was also partly supported by the Association Fran%E7aise contre les Myopathies (AFM, Nr. 15123).

