# Supplementary material for: Interspecies Translation of Disease Networks Increases Robustness and Predictive Accuracy
Source: PLoS Comput Biol. 2011 Nov 3;7(11):e1002258. doi: 10.1371/journal.pcbi.1002258 (PMC3207951; doi:10.1371/journal.pcbi.1002258)
Supplement: Text S1 — This supplementary material includes information on terminological definitions, protocols, gene lists, a set of primers used for validation study, and correlation analysis. (PDF) [file pcbi.1002258.s001.pdf]

SUPPLEMENTARY MATERIALS

Interspecies translation of disease networks increases robustness and predictive accuracy

Seyed Yahya Anvar[1,*], Allan Tucker[2], Veronica Vinciotti[2], Andrea Venema[1], Gert-Jan B van Ommen[1], Silvere M van der Maarel[1], Vered Raz[1] , Peter AC 't Hoen[1]

[1]Center for Human and Clinical Genetics, Leiden University Medical Center, P.O. Box 9600, 2300 RC Leiden, the Netherlands. [2]Center for Intelligent Data Analysis, School of Information Systems, Computing and Mathematics, Brunel University, Uxbridge, Middlesex, UB8 3PH, United Kingdom.

Correspondence: s.y.anvar@lumc.nl

Table S1 – Terminological definitions.

**Term**	**Definition**
Disease Module	Molecular pathway in which gene expression profiles are significant associated with the disease phenotype. Modules are described based on the current KEGG (Kyoto Encyclopedia of Genes and Genomes) annotation of molecular pathways.
Intraspecies Network	Gene network in which structural relationships among genes are based on the training with data from a single organism.
Interspecies Network	Gene regulatory network of which the structure holds a consensus across all species.
Sum Squared Error	The SSE measurement is the sum of the squares of the deviations between the measured expression values (or assigned disease phenotype) and the values predicted from the response variable which can be the class node (discrete variable), gene or gene transcript node (continuous variable). The identifier for the graph node is represented by *g* and the case id is represented by *i*. $$SSE_g = \sum_{i=1}^{n} (measured\ value_{g,i} - predicted\ value_{g,i})^2$$
Sensitivity	The probability of accurate prediction of cases with the disease-associated phenotype. $$Sensitivity = \frac{number\ of\ True\ case^{disease}}{total\ number\ of\ case^{disease}}$$
Specificity	The probability of accurate prediction of control cases without the disease-associated phenotype. $$Specificity = \frac{number\ of\ True\ case^{control}}{total\ number\ of\ case^{control}}$$
Confidence Score	The ratio of the number of times a link is found in a network structure to the maximum number of times the link can be found.<br>1. For the training set (species *A*): $$Confidence\ Score = \frac{number\ of\ times\ a\ link\ is\ found\ (n^{species\ A})}{total\ number\ of\ constructed\ networks\ on\ A}$$<br>2. For the independent test set (species *B*): $$Confidence\ Score = \frac{number\ of\ times\ a\ link\ is\ found\ (n^{species\ B})}{n^{species\ A} \times total\ number\ of\ constructed\ networks\ on\ B}$$
Robustness	The number of relationships found for genes from the disease module compared to those from random genes after applying different confidence thresholds.
Translatability	The likelihood of finding genes neighboring relatives that are selected as part of the intraspecies network structure during the phase of independent testing in the other species.
Naïve Dandelion	A class of Dandelion algorithm in which the networks are constructed on datasets derived from different organisms, where transcript expression levels for the same gene are averaged.
Exhaustive Dandelion	A class of Dandelion algorithm in which the structure of intraspecies networks are learnt on gene transcript level. This procedure involves a model-driven selection of the most probable homologous transcript isoform which is best translated across species.
Disease Domain	A sub-network structure associated with the class (disease) node which is defined based on the Markov blanket principle for the extension of the class node connectivity. This sub structure is composed of class node, its children, and its children's other parents that share the same level of confidence (≥0.1). A Markov blanket of the class node is the only knowledge needed to predict the disease phenotype.

Protocol S1 – Algorithm for Simulated Annealing Structure Learning.

Input: $t_0 = 10$, $maxfc = 1000$, D, $mode$, $netmap$

$fc = 0$, $t = t_0$, $t_n = 0.001$

$c = (t_n/t_0)^{1/maxfc}$

Initial *bn* to a Bayesian classifier with no inter-gene links

result = bn

oldscore = score(bn)

While *fc < maxfc* **do**

For each operator **do**

If *mode = 'train'*

Apply operator to *bn*

Else if *mode = 'test'*

Apply operator to *bn* **based on links available in** *networkMap*

End if

newscore = score(bn)

fc = fc + 1

dscore = newscore – oldscore

If *newscore > oldscore* **then**

result = bn

Else if $r(0,1) < e^{dscore/t}$ **then**

Undo the operator

End if

End for

t = t x c

End while

Output: *result*

Protocol S2 – Dandelion algorithm of interspecies construction of disease network

Input: $Species_{train}$, $\{Species_{test\ 1}, \ldots, Species_{test\ M}\}$, $train_{folds}$, $\{test_{folds\ 1}, \ldots, test_{folds\ M}\}$, $exhaustive_{T/F}$

For *k = 1* **to** $train_{folds}$

Learn $intraspeciesTranscript_{bn}$ using **Algorithm 1** on training folds of $Species_{train}$

Score $Scpecies_{train}$ $\{Nodes_{SSE}, Nodes_{STD}, Links_{Confidence}\}$

If *exhaustive = true*

Transform $intraspeciesTranscript_{bn}$ **to** $intrascpeciesGene_{bn}$

End if

Assess Disease Connection

If $intraspeciesGene_{bn}$ is not connected to *disease node* **then**

Drop $intrascpeciesGene_{bn}$

Else

Translate $intrascpeciesGene_{bn}$ to *networkMap*

For *i = 1* **to** *M*

Optimize and **Test** *networkMap* in $Species_{test\ i}$ using **Algorithm 1**

Score $Scpecies_{test\ i}$ $\{Nodes_{SSE}, Nodes_{STD}, Links_{Confidence}\}$

End for

End if

End for

Integrate $intraspeciesGene_{bn}$ using $Links_{Confidence}$ threshold of *0.1*

Output: $interspecies_{bn}$

Table S2 - Gene lists for independent tests and performance assessments.

**Proteasome and 30 Random Genes**		**100 Random Genes**		**70 Random Genes (not deregulated)**		**Ribosome**	
PSMD3	LOC643791	LOC644993	LOC651979	CPSF4L	WTAP	FAU	RPS6
PSMD12	C9orf79	LOC147710	OR4A47	LOC652683	CRTC2	RPSA	RPS7
PSMD11	MGRN1	PCDHB5	KCTD14	MME	LSM14B	RPL10A	RPS9
PSMD6	LOC653587	KIAA1688	CDK5RAP2	LOC653261	PRKG2	RPL3	RPS10
PSMD7	CNGA4	A4GALT	TMPRSS4	CD200R1	LUM	RPL3L	RPS11
PSMD13	OTOR	SFN	ADAMTS13	HSD11B1	PRUNE	RPL4	RPS12
PSMD14	GPR89A	BCL10	FRAS1	PDE4DIP	RPS3AP47	RPL5	RPS13
PSMD8	GPR89B	MSX2	SCUBE1	EEPD1	P2RX2	RPL6	RPS14
SHFM1	HAPLN4	SNRPB	LOC642855	KRTAP4-11	NAV1	RPL7	RPS15
PSMD4	LOC641994	HERC3	LOC442261	SLFN14	XRCC2	RPL7A	RPS15A
PSMD2	THBS2	HRASLS2	ZNF100	POU4F1	C17orf87	RPL8	RPS16
PSMD1	ZNF768	DLD	HDGFRP3	LOC442132	CACNA1I	RPL9	RPS17
PSMC2	KIAA1147	LOC649217	LOC642453	ST6GLA2	ELSPBP1	RPL11	RPS18
PSMC1	C19orf59	IGHG1	RHBDD1	ACTR3B	EPGN	RPL12	RPS19
LOC643668	BARHL2	GNPTAB	RSL1D1	PEF1	LOC650933	RPL13	RPS20
PSMC5	LOC400831	NOC4L	LOC652610	OGG1	HDX	RPL15	RPS21
PSMC6	HMGN4	PLD3	LOC646699	TAF9B	APOL3	RPL17	RPS23
PSMC3	TSSK4	LOC648974	KNDC1	LOC653421	CNOT4	RPL18	RPS24
PSMC4	RTKN2	GTPBP8	DACT3	LOC441347	PFAS	RPL18A	RPS25
PSMA6	RXRA	LIF	FLJ16369	FRMPD2	MAP3K14	RPL19	RPS26
PSMA2	MYL5	LOC440104	VIPR1	HSCB		RPL21	RPS27
PSMA4	UBTD1	WAC	COPS8	CHD1		RPL22	RPS27A
PSMA8	OR1J4	KALRN	NIF3L1	LOC645781		RPL23A	RPS28
PSMA7	TRAPPC5	UNC93A	PPAP2C	LOC729446		RPL24	RPS29
PSMA5	ADAM20	IFNAR1	LOC644431	FAM129C		RPL26	UBA52
PSMA1		NMT1	TCTE3	FAM90A15		RPL27	RPL14
PSMA3		LOC652750	TTF2	C1orf187		RPL30	RPL23
PSMB6		LOC653707	RPS7	HIPK2		RPL27A	RPL35
PSMB7		SLC26A9	ITGA8	XKR3		RPL28	RPL13A
PSMB3		ETFDH	CCAR1	RAB2A		RPL29	RPL36
PSMB2		ADAM23	PDCD10	FOXR1		RPL31	MRPL13
PSMB5		FBXO9	LOC651400	CD72		RPL32	RPS27L
PSMB1		LOC643089	CDC42BPG	TRAF4		RPL34	RPL26L1
PSMB4		ATP5D	SP2	NCAN		RPL35A	C15orf15
PSME1		CST6	LOC649432	HRC		RPL36AL	RPL10L
PSME2		RPL11	LOC732093	LOC643577		RPL37	RPL22L1
PSME3		FAM47B	TMEM165	AKR7A2P1		RPL37A	RSL24D1P11
PSME4		LHFPL4	LHCGR	PLK2		RPL38	
POMP		MGC42105	SPAG7	RABL2B		RPL39	
PSMF1		STOX2	INOC1	CLGN		RPL41	
IFNG		FRMD5	OR2T10	LRRC49		RPL36A	
PSMB9		CHL1	DEPDC5	CHORDC1		RPLP0	
PSMB10		UNQ830	ADAD1	KRT18P51		RPLP1	
PSMB8		STCH	LOC339529	OR13G1		RPLP2	
PSMB11		B4GALNT3	FZD9	CCL21		RPS2	
AKR1CL1		SUMO2	CD46	LRFN2		RPS3	
CHRNA5		C20orf30	JARID1B	SLC35A5		RPS3A	
UNC13B		CNIH3	DUX4	RDH12		RPS4X	
DES		DBX2	DPPA4	FAM154B		RPS4Y1	
STT3A		GSTM5P1	YSK4	LOC388948		RPS5	

Table S3 – The list of primers that were used for qPCR validation study in IM2 cell model of OPMD.

Gene	FW Primer Sequence	RV Primer Sequence
RPN11 (Psmd14)	CACCTGAACAGCTGGCAATA	GAGCATTGGGAACGAAGAAG
RPN15 (Shfm1)	AGCACGGCTACAAGATGGAG	TGAACCAAAAAGATTAAATCAAAACA
RPT3 (Psmc4)	ACCTCAGACCAGAAGCCAGA	CACCACACGGATAAATGCAG
β2 (Psmb7)	GCACTACCGCTGTCCTCACCG	AGGGGTGGTATGCACCCCGAG
β5 (Psmb5)	CGGTCGCAGCAGCCTCCAAA	GCATACACGGAGCCAGAGCCC
PA28α (Psme1)	AAGCCAAGGTGGATGTGTTC	GGGTACTGGGATGTCCAATG
PA28β (Psme2)	CCTGGAGAGTGAAAGCGAAA	GTCATCAGCCTCCTGGAAAA
β2i (Psmb10)	ATTTGCTCCTGGAACCACAC	CCACTTCATTCCACCTCCAT
ACTA1 (Acta1)	CGAGGTATCCTGACCCTGAA	AGGTGTGGTGCCAGATCTTC
mHPRT	CGTCGTGATTAGCGATGATG	TTTTCCAAATCCTCGGCATA

Table S4 – Correlation between the expression profiles of genes selected from the interspecies disease domains.

			Interspecies	Human		Mouse		Drosophila	
Gene A	Gene B	Train Set	Confidence	Correlation Score	*P*-value	Correlation Score	*P*-value	Correlation Score	*P*-value
PA28α	RPN1	Human	Strong	0.6059	2.80E-03	0.8650	8.50E-11	-0.3762	2.37E-02
PA28α	RPN15	Human	Strong	-0.0466	8.37E-01	0.7521	4.50E-07	0.4801	3.03E-03
PA28α	RPN8	Mouse	Strong	-0.0670	7.57E-01	0.9245	1.58E-14	0.4658	4.20E-03
PA28α	RPT3	Mouse	Strong	0.5984	3.26E-03	0.7944	3.42E-08	0.4491	6.00E-03
PA28α	α3	Human	Strong	-0.5234	1.24E-02	0.8988	1.24E-12	0.5498	5.14E-04
RPN10	RPN3	Mouse	Moderate	0.5450	8.72E-03	0.6400	6.07E-05	0.5503	5.06E-04
RPN10	RPN6	Mouse	Moderate	0.6300	1.68E-03	0.8634	1.00E-10	0.8180	1.12E-09
RPN10	RPN9	Mouse	Strong	0.3338	1.29E-01	0.8760	2.46E-11	0.8545	3.36E-11
β3	RPN12	Mouse	Moderate	0.4635	2.98E-02	0.8653	8.15E-11	0.5090	1.52E-03
β3	RPN7	Mouse	Moderate	-0.2445	2.73E-01	0.9386	7.17E-16	0.6361	3.05E-05
β3	RPT3	Drosophila	Strong	0.4274	4.72E-02	0.8652	8.32E-11	0.5855	1.76E-04
β3	α3	Mouse	Moderate	0.0910	6.87E-01	0.9456	1.15E-16	0.8367	2.07E-10
β3	α4	Drosophila	Strong	0.2359	2.91E-01	0.9378	8.65E-16	0.7959	6.56E-09
β5i	α6	Mouse	Strong	-0.5980	3.28E-03	0.6593	3.01E-05	0.6393	2.70E-05
β5i	β1i	Mouse	Moderate	0.6729	6.00E-04	0.9053	4.63E-13	-0.1952	2.54E-01
β5i	β4	Mouse	Strong	0.1983	3.76E-01	0.7679	1.83E-07	0.6416	2.48E-05
